# Polyglycolic Acid Sheet Application to Refractory Delayed Bleeding After Rectal Endoscopic Submucosal Dissection: A Case Report

**DOI:** 10.1002/deo2.70231

**Published:** 2025-10-25

**Authors:** Yoshiko Nakano, Katsutoshi Kuriyama, Yasuhiro Watanabe, Shin'ichi Miyamoto

**Affiliations:** ^1^ Department of Gastroenterology National Hospital Organization Kyoto Medical Center Kyoto Japan

**Keywords:** delayed bleeding, endoscopic submucosal dissection, fibrin glue, polyglycolic acid sheet, rectum

## Abstract

A man in his 50s underwent endoscopic submucosal dissection (ESD) for a rectal tumor measuring 28 mm. He was not taking any antithrombotic medication. On the third, 11th, 18th, and 24th days after the procedure, bleeding was observed from different areas of the ulcer bed margin and was managed with hemostatic forceps. Despite epithelization and ulcer healing, delayed bleeding recurred, with the fourth episode being severe, marked by significant bleeding and a decrease in serum hemoglobin level. Although clip closure of the ulcer bed and the application of PuraStat were attempted, they failed to prevent recurrent bleeding. Therefore, polyglycolic acid (PGA) sheets were applied to the ulcer bed during the fourth episode. Four days later, PGA sheets remained on the ulcer bed without any signs of bleeding, and healing continued. No further bleeding episodes occurred. The fact that continuous hemostasis was achieved after application of the PGA sheet without complications suggested that this approach may be an option in future cases of refractory delayed bleeding after rectal ESD.

## 1 Introduction

Endoscopic submucosal dissection (ESD) for superficial gastrointestinal neoplasms is now more widely practiced. Complications associated with ESD include intraoperative perforation, bleeding, delayed perforation, and postoperative bleeding. The incidence of postoperative bleeding is lower with colorectal ESD than with gastric ESD [1, 2, 3]. We encountered a case of refractory postoperative bleeding that recurred after rectal ESD. Although various interventions were applied, rebleeding repeatedly occurred. Ultimately, polyglycolic acid (PGA) sheets with fibrin glue proved to be effective. We suggest that this approach could be a viable option for refractory delayed bleeding following colorectal ESD.

### 1.1 Case Report

**FIGURE 1:**
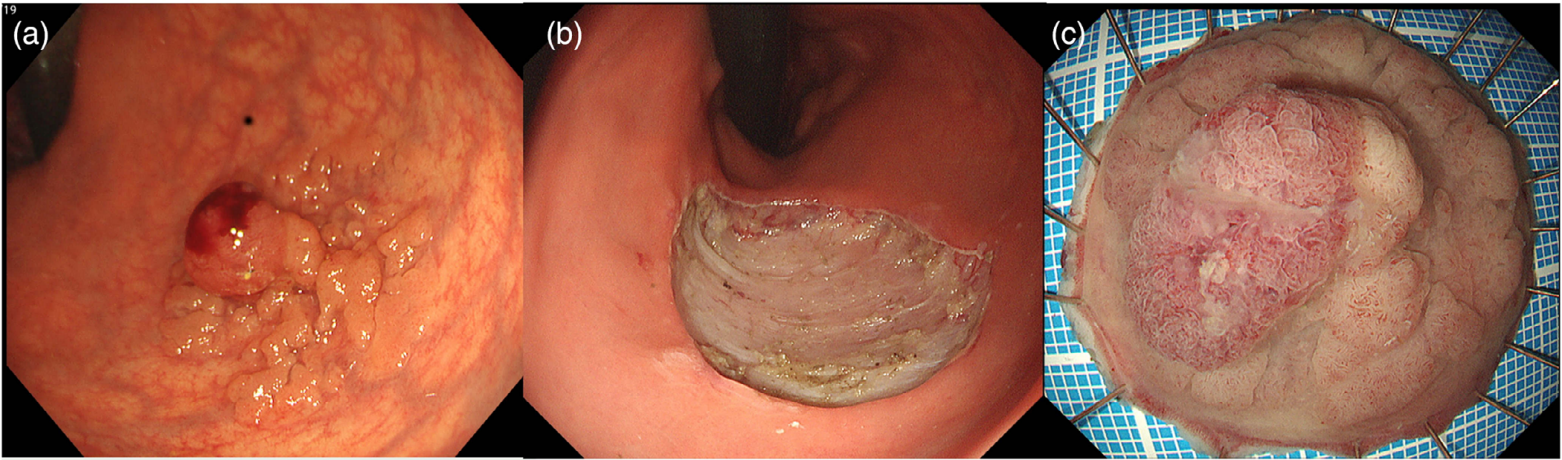
(a) A laterally spreading granular‐type tumor measuring 28 mm in the upper rectum. (b) Endoscopic submucosal dissection was performed. (c) Resected specimen. The pathological diagnosis was high‐grade tubulovillous adenoma, with negative surgical margins.

**FIGURE 2:**
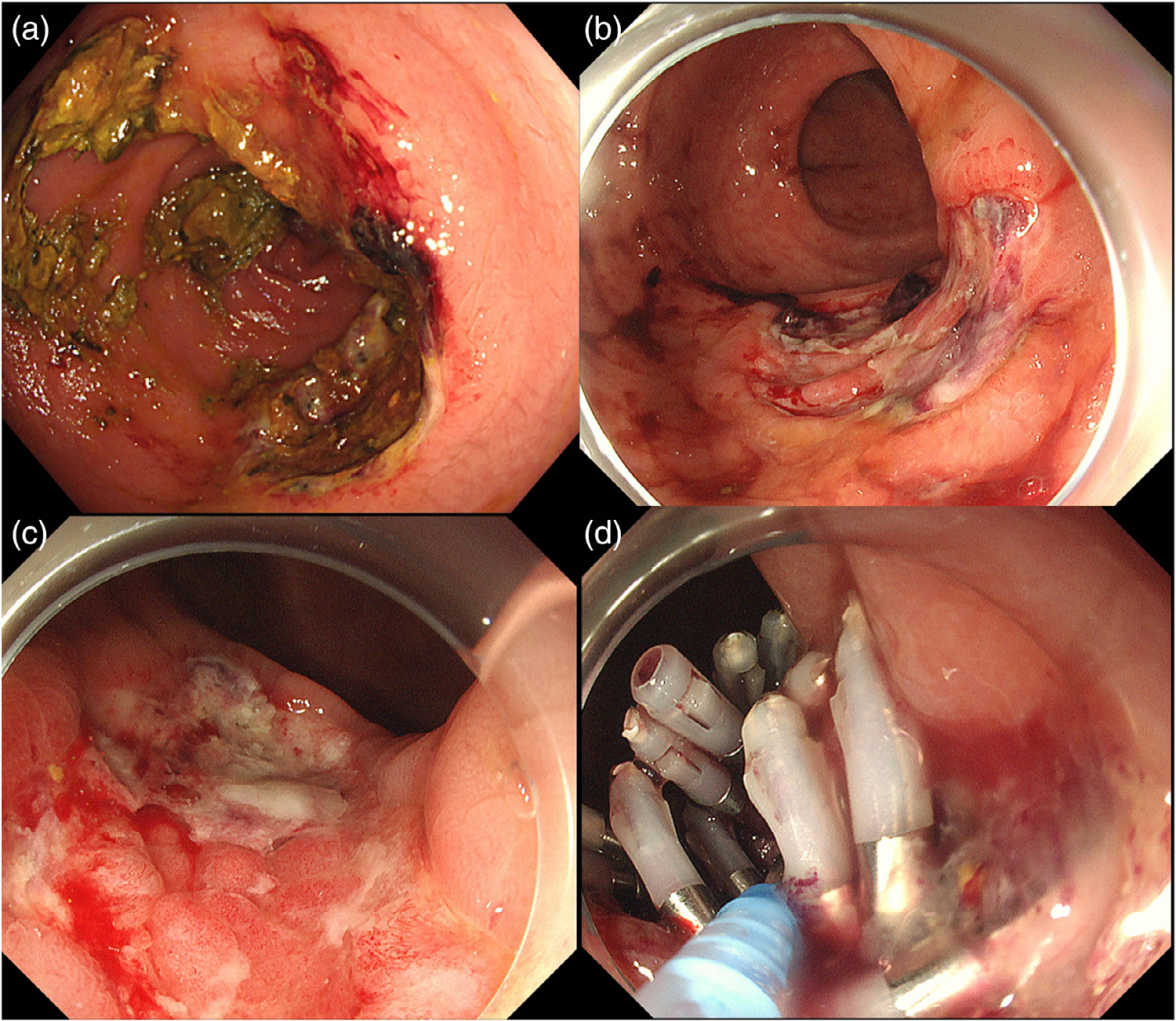
(a) Delayed bleeding on the third day after the procedure, which was treated with hemostatic forceps. (b) Delayed bleeding on the 11th day after the procedure, which was at a different site from that of previous bleeding and was treated with hemostatic forceps. (c) Delayed bleeding on the 18th day after the procedure. Although more than half of the ulcer bed had epithelialized and was healing. A small amount of bleeding was observed at the edge of the epithelialized area and was treated with hemostatic forceps. (d) After cauterization with hemostatic forceps, clip closure of the ulcer bed was attempted to prevent recurrent delayed bleeding. However, complete closure was difficult due to the hardened edges of the ulcer, and, thus, PuraStat was applied to the ulcer.

**FIGURE 3:**
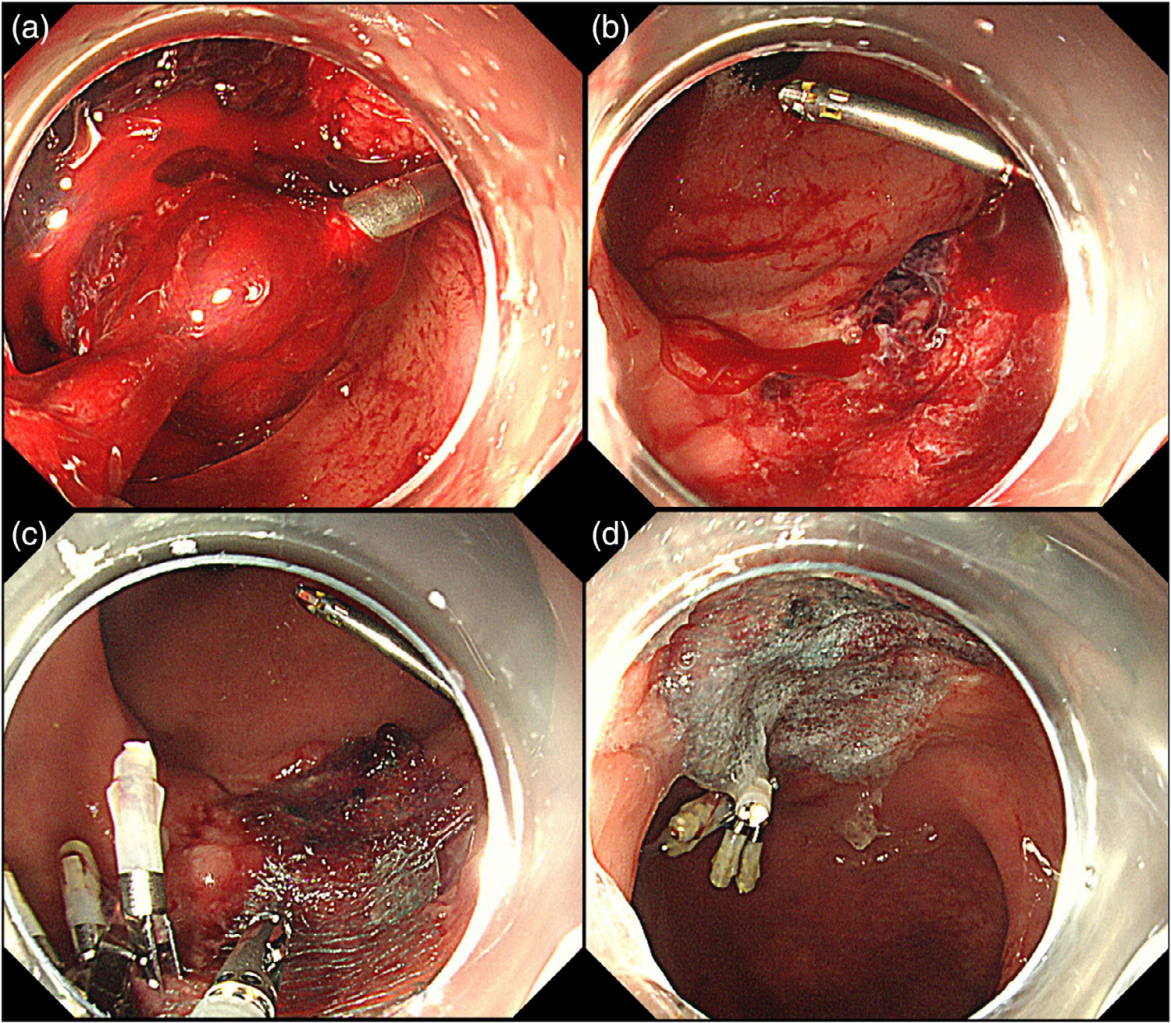
(a) Delayed bleeding with a large clot in the rectum on the 24th day after the procedure. (b) Active bleeding was observed from the ulcer bed near the anal verge. (c, d) After cauterization with hemostatic forceps, polyglycolic acid (PGA) sheets cut into 1 cm^2^ pieces were applied to cover the ulcer bed, and fibrin glue was applied.

**FIGURE 4:**
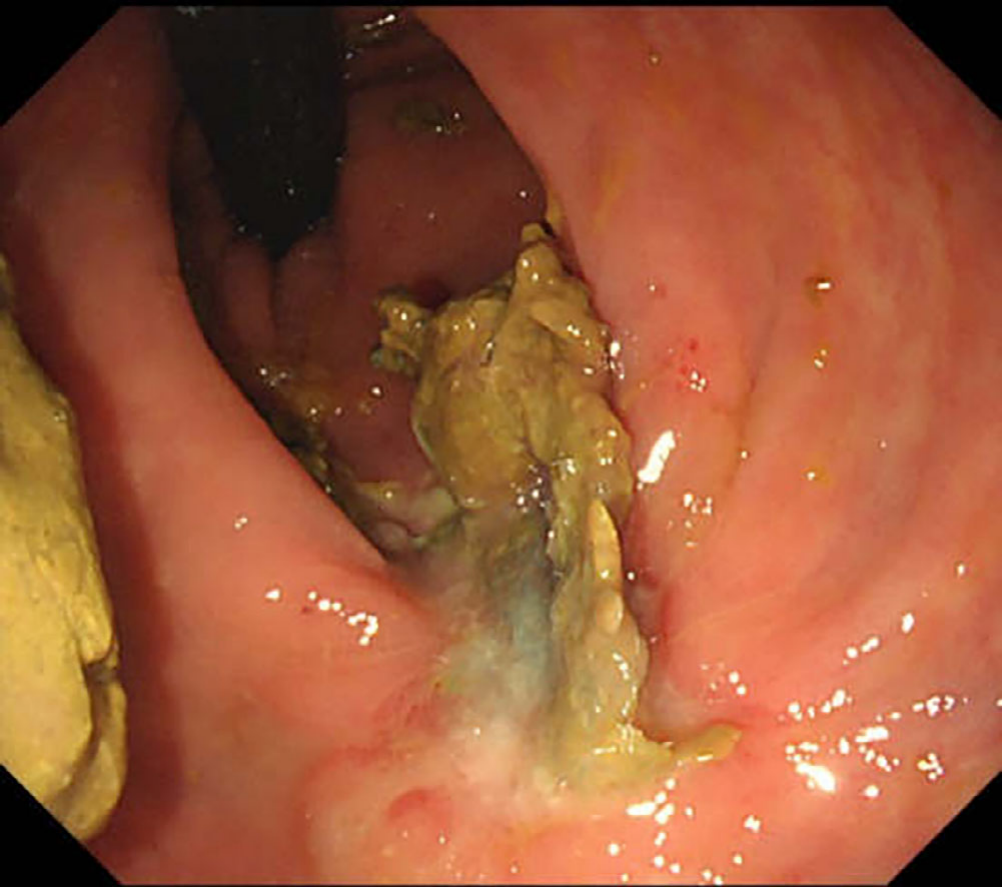
On the 28th day after the procedure, polyglycolic acid (PGA) sheets were confirmed to remain on the ulcer bed, with no exposed blood vessels or present bleeding, and ulcer healing was progressing.

A male in his 50s presented to a nearby clinic with blood in his stools during bowel movements and was referred to our hospital for further examination. He had a history of second‐degree atrioventricular block and paroxysmal supraventricular tachycardia and was not taking any antithrombotic medication. Colonoscopy revealed a laterally spreading granular‐type tumor measuring 28 mm in the upper rectum (Figure 1a). ESD was performed, and the tumor was successfully removed en bloc without any complications (Figure 1b). The tumor was pathologically diagnosed as a high‐grade tubulovillous adenoma, with negative surgical margins (Figure 1c). The patient had a favorable recovery; however, on the third day after the procedure, a small amount of hematochezia was noted. Colonoscopy showed coagulation and oozing bleeding at the ulcer bed margin (Figure 2a), which was treated with hemostatic forceps. The patient was subsequently discharged. On the 11th day after the procedure, the patient again presented with a small amount of blood in his stools. Colonoscopy revealed coagulation and oozing bleeding at the oral side of the post‐ESD ulcer bed (Figure 2b), which was different from the previous bleeding site and was also treated with hemostatic forceps. However, on the 18th day after the procedure, the patient again presented with a small amount of hematochezia. Although more than half of the ulcer bed had epithelialized and was healing, a small amount of bleeding was observed at the edge of the epithelialized area (Figure 2c). Cauterization with hemostatic forceps was performed, and clip closure of the ulcer bed was attempted to prevent recurrent delayed bleeding; however, complete closure was difficult because the edges of the ulcer were firm during the healing process. We applied PuraStat (3D Matrix, Tokyo, Japan), a novel self‐assembling peptide hemostatic hydrogel (Figure 2d). On the 24th day after the procedure, the patient presented with a large amount of bloody stool and was re‐examined. His serum hemoglobin level decreased from 15.9 to 13.9 g/dl. Colonoscopy revealed a large clot in the rectum (Figure 3a), and active bleeding was observed from the ulcer bed near the anal verge (Figure 3b). Previously placed clips had fallen off. The PGA sheet was regarded as the only remaining option, as hemostasis with coagulation forceps was unsuccessful, complete clip closure of the ulcer bed was not feasible, and PuraStat application was ineffective. After hemostasis was achieved using hemostatic forceps, PGA sheets with fibrin glue were applied to cover the ulcer bed (Figure 3c,d). We positioned the ulcer bed on the gravity‐dependent upper side to minimize submersion in fluid. After removing the forceps cap of the endoscope, we carefully delivered 1 cm^2^ pieces of PGA sheet to the ulcer bed one by one, trying to keep them as dry as possible, and spread them out. After applying four 1 cm^2^ PGA sheets, the ulcer bed was almost completely covered, so we then sequentially sprayed fibrin glue (Beriplast P Combi‐Set, CSL Behring Pharma, Tokyo, Japan; or BOLHEAL, KM Biologics Ltd., Kumamoto, Japan) over the area using an endoscopic catheter. The sheets were delivered carefully, as dry as possible, and spread evenly to prevent clumping of the sheets, which could increase the risk of detachment from the ulcer bed. The procedure time from the delivery of the PGA sheet to the completion of fibrin glue application was 25 min. The patient was admitted, fasted, and then resumed eating the following day. On the 28th day after after ESD, colonoscopy confirmed that PGA sheets remained on the ulcer bed, with no exposed blood vessels or present bleeding, and ulcer healing was progressing (Figure 4), compared to the change between the 18th day and 24th day. There were no further episodes of bleeding.

## 2 Discussion

The present case experienced four episodes of bleeding after rectal ESD, with the fourth episode occurring 24 days after treatment and involving the largest amount of bleeding despite ongoing healing of the ulcer bed. Therefore, the ulcer bed was highly prone to bleeding. Although hemostatic forceps achieved temporary hemostasis, rebleeding occurred, and complete clip closure of the ulcer bed was difficult due to hardening of the ulcer's edges over time after ESD. After the application of PGA sheets with fibrin glue, there was no rebleeding.

The post‐procedure bleeding rate of gastric ESD was previously reported to be 4.4% by a Japanese multicenter prospective cohort study [1], while that of colorectal ESD was lower at 1.7%–2.2% [3, 4]. However, recurrent refractory delayed bleeding occurs in some cases.

Risk factors for post‐ESD bleeding in the colorectum include large tumors (≥30 mm), the location in the rectum, and the use of antiplatelet/anticoagulant agents, except for aspirin alone [5]. In the present case, the identified risk factor was the location in the rectum. The higher incidence of rectal bleeding has been attributed to several factors, including the abundance of large blood vessels, increased abdominal pressure during bowel movements, and direct contact with solid stools. A number of endoscopic closure techniques have recently been reported in addition to conventional clips. Prophylactic clip closure after colorectal ESD in patients receiving anticoagulant therapy effectively prevented delayed bleeding after the procedure [6]. However, a recent multicenter retrospective cohort study in Japan, including 34,455 colorectal ESD cases from 47 Japanese institutions, showed that although complete closure significantly reduced the risk of delayed bleeding among patients taking direct oral anticoagulants for right‐sided lesions, no risk reduction was observed for left‐sided or rectal lesions [7]. Based on this finding and repeated bleeding in the present case, even after the progression of ulcer healing, it remains unclear whether post‐ESD bleeding may have been prevented if prophylactic clip closure had been performed.

PGA is an absorbable reinforcement material that was originally used in surgical fields and has recently been introduced to the endoscopic field to prevent delayed perforation after ESD and close iatrogenic perforations and fistulas [8]. There is a report that an intractable acute hemorrhagic rectal ulcer that required 19 endoscopic hemostasis procedures over a 2‐month period was successfully treated by the application of PGA sheets with fibrin glue [9]. Tsuji et al. reported that endoscopic tissue shielding using PGA sheets to cover post‐ESD wounds in 10 colorectal cases resulted in no procedure‐related adverse events and may help reduce postoperative complications [10]. In the present case, continuous hemostasis with ulcer healing was observed within four days after PGA sheet application, suggesting that PGA sheets contributed to wound healing by acting as a scaffold for tissue regeneration. The effectiveness of PGA sheets in refractory post‐ESD bleeding is likely attributable to both the protection of the ulcer bed from external stimuli such as fecal matter and the promotion of ulcer healing. However, the application of PGA sheets is not necessary in all cases but only for refractory post‐procedure bleeding, because the risk of bleeding after colorectal ESD is relatively low, and performing it universally would be very inefficient in terms of time and cost‐effectiveness. If prophylactic hemostasis is to be performed in cases at high risk of delayed bleeding, using clips—an approach most endoscopists are familiar with—is likely the first choice.

However, in cases where clip closure is difficult, the use of a PGA sheet can be an option. PGA is best reserved as a last‐resort option when conventional hemostatic methods are infeasible or ineffective. The present case suggests that rectal ulcers may experience recurrent refractory bleeding. In these cases, PGA sheets combined with fibrin glue appear to be an option for managing refractory bleeding.

## Author Contributions

Conceptualization and resources: Yoshiko Nakano; The physicians in charge of the patient: Yoshiko Nakano, Katsutoshi Kuriyama, and Yasuhiro Watanabe; Writing–original draft: Yoshiko Nakano; Writing–review & editing: all authors.

## Conflicts of Interest

The authors declare no conflicts of interest.

## Funding

The author has nothing to report.

## Ethics Statement

The patient provided informed consent for the publication of this case report.
